# Comparing modeling approaches for assessing priorities in international agricultural research

**DOI:** 10.1093/reseval/rvx044

**Published:** 2019-09-17

**Authors:** Athanasios Petsakos, Guy Hareau, Ulrich Kleinwechter, Keith Wiebe, Timothy B. Sulser

**Affiliations:** 1 Social and Nutrition Sciences Division, International Potato Center (CIP), Avenida La Molina 1895, Lima 12, Peru and; 2 Environment and Production Technology Division, International Food Policy Research Institute (IFPRI), 2033K St. NW, Washington, DC 20006, USA

**Keywords:** priority setting, foresight analysis, economic surplus model, international agricultural research

## Abstract

This article examines how the estimated impacts of crop technologies vary with alternate methods and assumptions, and also discusses the implications of these differences for the design of studies to inform research prioritization. Drawing on international potato research, we show how foresight scenarios, realized by a multi-period global multi-commodity equilibrium model, can affect the estimated magnitudes of welfare impacts and the ranking of different potato research options, as opposed to the static, single-commodity, and country assumptions of the economic surplus model which is commonly used in priority setting studies. Our results suggestthatthe ranking oftechnolo- gies is driven by the data used for their specification and is not affected by the foresight scenario examined. However, net benefits vary significantly in each scenario and are greatly overestimated when impacts on non-target countries are ignored. We also argue that the validity of the singlecommodity assumption underpinning the economic surplus model is case-specific and depends on the interventions examined and on the objectives and criteria included in a priority setting study.

## 1. Introduction

There is a growing demand from public and private donors and other decision makers for more efficient spending of resources used in agricultural research. At the same time, tightened budgets have led agricultural research organizations to formalize priority setting approaches for the identification of research activities with the highest possible impact in terms of economic efficiency, poverty alleviation, and other institutional, social, and environmental objectives, to inform decisions on the optimal allocation of research funds. Priority setting increases the credibility and objectivity of the decisions taken at the various institutional levels (institutes, programs, and projects) and offers a systematic way of planning and managing research which is consistent with informed scientific opinion and stakeholder needs (Bantilan and Keatinge 2007).

Various qualitative and quantitative approaches have been used for priority setting, including scoring methods, congruence rules, benefit-cost analysis, economic surplus models, and multi-objective programming, each with distinct advantages and drawbacks compared to the others (Schumway 1977; Braunschweig 2004). Priority setting can be limited to the subjective ranking of the various research options using expert judgement, or it may involve formal ex *ante* impact assessment to quantify the potential benefits of alternative technologies. In the latter case, an economic surplus model combined with discounted benefit-cost measures is probably the most common method (Alston et al. 1998). In its simplest form, the economic surplus model is a comparative static representation of the supply and demand equilibrium for a single market (commodity). It can estimate welfare changes for producers and consumers brought about by policies or technological innovations. Extensions to the basic model include price and technology spillovers among countries or regions connected via trade (Davis et al. 1987; HarvestChoice 1995), while Geographical Information Systems have also been proposed for identifying similar agroecologies in neighboring regions to quantify the direction and intensity of these spillover effects (You and Johnson 2010).

The economic surplus model has been widely applied in *ex ante* impact assessment studies to inform decisions for defining priorities, both in national research programs (Dey and Norton 1992; Mutangadura and Norton 1999), and in International Agricultural Research Centers (Briones et al. 2008; Hareau et al. 2014). One important limitation of the approach is that it offers a static representation of the modeled commodity market and thus ignores the socioeconomic and biophysical dynamics which can affect the expected impact of a given intervention. Although some of its implementations allow for an explicit representation of dynamics in production and consumption (HarvestChoice 1995), the economic surplus model is not able to analyze well-structured foresight scenarios, like those proposed by the Intergovernmental Panel on Climate Change (IPCC) which describe plausible future pathways in population, income, climate, and other drivers of change. Furthermore, with some few exceptions (Davis et al. 1987), the analysis of agricultural research priorities at the regional or international level with economic surplus models either ignores welfare impacts on horizontally related markets (countries not targeted for the release of new technologies) or does so aggregately, probably because it is difficult to estimate the individual demand and supply curves in each country (Alston et al. 1998). Finally, the economic surplus model cannot directly account for the effects of technological innovations on multiple commodity markets. Instead, crossprice effects are implicitly represented in the assumed supply and demand elasticities of the commodity examined (Omamo et al. 2006). In this regard, several studies have used partial or general equilibrium models to estimate the direct and indirect welfare impacts of new technologies (Rosegrant et al. 2014; Islam et al. 2016), yet not explicitly for ranking specific investment options. One possible reason is that multi-commodity equilibrium analyses seem to be beyond the scope of priority setting exercises (Byerlee 2000).

Given the aforementioned limitations, this article seeks to provide a quantitative assessment of whether the relaxation of the related assumptions in the economic surplus approach adds value to a priority setting exercise and can therefore justify a shift from the standard quantitative paradigm in studies aiming to inform research prioritization. More precisely, we examine how the simulated dynamics of a quantitative foresight scenario analysis can influence the returns to investment and the ranking of different crop research options, and to what extent the consideration of other countries can affect the welfare results, as opposed to focusing on target countries only. We also look at multiple commodity markets to assess the importance of cross-price effects which are ignored under the simple economic surplus model approach.

Our study builds on the analysis of research priorities for potato which was carried out by the CGIAR Research Program (CRP) on Roots, Tubers, and Bananas (RTB) (Hareau et al. 2014) and constitutes an analytical example of using economic surplus models for informing priority setting decisions in international agricultural research. In this article, we replicate the RTB exercise and then estimate the expected global welfare impacts of four of the same potato research options under different foresight scenarios with the IMPACT model (International Model for Policy Analysis of Agricultural Commodities and Trade), which is a recursive dynamic multi-commodity partial equilibrium representation of the global agricultural sector (Robinson et al. 2015), developed at the International Food Policy Research Institute (IFPRI). For examining the role of dynamics, we compare the welfare results from foresight scenarios simulated with IMPACT against a counterfactual which is created by keeping constant all model parameters that are intrinsically dynamic, like population, income, and climate impact on yields. This counterfactual therefore represents a static scenario of technology adoption and serves as a proxy for the economic surplus model, since it only considers a research-induced increase in crop yields which finally leads to an endogenously calculated outward shift of the supply function, ceteris paribus. By also looking at different result aggregation levels (target countries and global) and the magnitude of cross-price effects, we examine how the underlying assumptions of the economic surplus model can influence the results of a priority setting study.

In the next section, we introduce the IMPACT model and present the four potato research options compared in this article. In Section 3 we explain the approach for simulating a productivity shock with IMPACT and detail the assumptions behind the different foresight scenarios considered. Results are presented in Section 4, followed by a discussion in Section 5 on implications of our findings for studies aiming to inform research prioritization.

## 2. Materials and methods

### 2.1 The IMPACT partial equilibrium agricultural sector model

IMPACT is an integrated modeling framework which combines economic, crop, livestock, and water models designed for the analysis of future developments of global agricultural production, demand, trade, and prices up to 2050. The model has been used extensively in analyses related to projections of global, regional, and national food supply and demand (Pinstrup-Andersen et al. 1997; Huang et al. 1999; Pandya-Lorch and Rosegrant 2000), commodity-specific analyses (Scott et al. 2000), or analyses of issues related to the agricultural sector, such water scarcity (Rosegrant and Cai 2001) and climate change (Nelson et al. 2010; Islam et al. 2016). IMPACT uses FAOSTAT and other data, and its projections are based on regional and global scenarios that draw on the fifth assessment report of the IPCC. A detailed description of the IMPACT modeling framework and underlying equations can be found in Robinson et al. (2015).

The food production module in IMPACT comprises 62 agricultural commodities and distinguishes 159 geopolitical regions and 154 water basins globally, which combine to 320 geographic ‘food production units’ (FPUs). Crop production takes place at the FPU level and in modeling terms is defined as the product of response functions for crop yields and harvested areas. Cropland is divided between irrigated and rainfed areas, and the crop share allocations are determined by a market which simulates the equilibrium between total land supply and the area demanded by each crop. Yields depend on exogenous trend parameters, input and crop prices, and possible water stress or climate-induced shocks. On the demand side, a set of separate functions is used to represent different demand components for each country and commodity, namely, food, feed, biofuels, crush demand for oilseeds, and other uses, which add up to total demand for any single crop. Whereas all demand types are a function of the commodity’s own price, demand for food additionally depends on the prices of other competing commodities (through cross-price elasticities), but also takes into account assumed changes in population and per capita income.

The individual regions for which supply and demand is calculated are connected to each other via trade. Net trade adds to domestic production to finally equilibrate domestic supply and demand. For each year simulated by the model, global demand and supply for every commodity are brought into equilibrium and determine an endogenous world market price which leads to global market clearing. The domestic producer and consumer prices for all commodities in all regions are derived from this world equilibrium price. When an exogenous shock is applied to the model, like increases in crop productivity as done in this study, the world market price adjusts to those changes to establish new market equilibrium and produce a new set of country-level prices, demand, supply, and trade (Scott et al. 2000).

IMPACT also includes a post-simulation module to estimate the welfare impacts of a given intervention scenario in relation to a reference (baseline) scenario. The estimations comprise producer surplus, consumer surplus, and net welfare effects to the agricultural sector. Further, the costs of a particular intervention, if available, can be taken into account to calculate the internal rate of return (IRR) as a measure of returns on investment. Consumer surplus is estimated by using the stored equilibrium point and the model’s own price demand elasticities to calculate a slope and price intercept parameter to create a linear demand function for each commodity. In contrast, estimation of producer surplus is not straightforward because supply in IMPACT is modeled as the product of yield and area response functions and not through a traditional supply curve which reflects a producer’s marginal cost curve. For this reason, a synthetic supply curve is created which expresses yields and harvested areas as a function of crop prices. Such supply curves for each commodity are created for every land type (irrigated, rainfed) in each FPU and are then aggregated at the national level.^1^

### 2.2 Proposed potato technologies

The research options (technologies) analyzed are (1) improved potato seed systems, (2) potato varieties resistant to bacterial wilt, (3) virus-resistant potato varieties, and (4) varieties resistant to late blight.^2^ These technologies were previously examined in the RTB analysis of research priorities and were identified by RTB via a multistage participatory process (Kleinwechter et al. 2014). For each research option, specific target countries in Africa, Asia, and Latin America were also identified. Their selection was based on the current distribution of the constraints addressed by the different research options and on considerations about current and future target geographical regions for international potato research for development, as carried out by RTB, the International Potato Center (CIP), and partners.

The quantification of every research option in each target country was based on expert judgment and consists of assumptions regarding the probability of research success, the expected adoption rates, and the expected changes in yield and input costs.^3^ An important note is that the maximum adoption rates and the probability of success should ideally be defined as empirical distributions (Alston et al. 1998). However, the RTB priority assessment study only provides country-specific point estimates for both parameters, elicited from expert workshops, and considers a ‘lower’ and a ‘higher’ adoption scenario to partially account for the uncertainty on the adoption process. To simplify the analysis, in this article we do not assume any variability for either the maximum adoption rates or the probability of success. All adoption data come from the ‘higher’ adoption scenario, and we define the probability of success as the mean of the odds for achieving the research objectives.

Further assumptions were made on the costs of research, development, and dissemination. Annual costs for research and development are an estimation of costs incurred by CIP and by the national agricultural research systems. For the dissemination cost, a fixed figure per hectare is assumed for the marginal area of adoption. This cost depends on the type of technology analyzed. Varietal technologies are assumed to require an investment of US$50 per hectare of finally adopted area, while more knowledge-intensive technologies, e.g. seed systems interventions analyzed herein, are assumed to require US$80 per hectare. Table 1 provides a summary of the technologies, while Sections 2.1.1-2.1.4 give a more detailed description for each research option and its parameterization as an adoption scenario. Detailed lists with the target countries and parameter values for each research option are provided as Supplementary Material.

**Table 1:** Summary oftechnology scenarios

Technology parameters	Improved seed systems	Bacterial wilt-resistant varieties	Virus-resistant varieties	Late blight-resistant varieties
Research lag (years)	3	10	2	2
Adoption lag (years)	5	10	10	10
Countries targeted^a^	27	22	27	32
Adoption ceiling (%)	3-20	10-60	15-40	10-60
Total annual R&D costs (million US$)	8	4	8	16
Dissemination costs (US$/ha)	80	50	50	50
Maximum expected yield change (%)	20	10-30	40	12-32
Production cost change (%)	20	0	-5	—2 to —5
Probability of success (%)	60-80	50	70	80

a See Supplementary Data for country-level specifications.

*Source:* Hareau et al. (2014).

#### 2.2.1 Seed systems: Improving seed production and distribution

Poor seed quality is a major constraint to potato yields in many regions (Gildemacher et al. 2009; Haverkort and Struik 2015). The improvement of potato seed production and distribution through the development and implementation of improved seed systems reduces yields losses from seed-borne diseases and improves farmers’ access to seed potato tubers of higher physical and physiological quality. The quantified impacts of the improved seed systems technology modeled here across 27 target countries are assumed to include an increase in potato yield of 20% and a rise in production costs by the same percentage as a result of the higher cost for quality seed and the increased use of other inputs. The research lag was set to 3 years, reflecting the investment that CIP has undertaken in previous years to develop methods and approaches for providing quality seed materials to farmers. Improved potato seed systems therefore represent a relatively well-developed technology which requires only some adjustment to local conditions in the target countries. Maximum adoption rates vary across the target countries from 3 to 20%, while the probability of research success is between 60 and 80%, based on the experience of CIP in seed systems work in the respective countries.

#### 2.2.2 Potato varieties resistant to bacterial wilt

Potato varieties resistant to bacterial wilt aim at reducing yield losses caused by the pathogenic bacterium *Ralstonia solanacearum,* which is a major biotic constraint in many developing countries, particularly in Africa (Gildemacher et al. 2009) and in the Andean region (Salazar 2006). The improved varieties are expected to increase yields in target countries by 10-30%, based on expert opinions about the incidence of the disease in each country. Since no chemical treatment is available for bacterial wilt, adoption is not expected to bring about input use changes, and thus production costs are assumed to remain constant. The technology has a research lag of 10 years, which is the longest among all four research options, since breeding efforts targeted at this trait need to be re-initiated. The probability of research success is assumed to be 50% because the development of bacterial wilt-resistant varieties requires more upstream research, and success is therefore more uncertain than for the other technologies considered.

#### 2.2.3 Virus-resistant potato varieties

Viruses are considered the most important biotic constraint for seed potato. Virus-resistant potato varieties aim at reducing yield losses caused by potato virus diseases, namely, potato virus Y, potato leaf roll virus, potato virus S, and potato virus X. Although some virus resistance has already been achieved, this technology aims at introducing even higher levels of virus resistance in future potato varieties. The impact pathway of virus-resistant potato varieties partially overlaps with the seed system technology in the development of high-quality virus-free potato seed. The technology further assumes high levels of virus resistance during the crop’s entire life cycle, and therefore it is expected to lead to the highest yield increases of all four options assessed. In addition, a small reduction in production costs of 5% across all countries is also assumed. This reflects potentially lower costs for seed replacement, since virus-resistant varieties are less prone to seed degeneration, and also lower labor costs due to reduced need for roguing infected plants. The development of virus-resistant varieties is rather complex, and therefore the estimated probability of success is assumed to be 70%. Since varieties with lower degrees of virus resistance are already available at CIP, the diffusion of the technology can start after a comparatively short research lag of only 2 years.

#### 2.2.4 Late blight-resistant potato varieties

Yield losses from late blight (*Phytophtora infestans*) are still considered to be the most important biotic constraint on potato production worldwide (Birch et al. 2012). Breeding for late blight-resistant varieties at CIP began in 1975, and today it is the primary trait of one of the two major breeding populations of the Center (the other being virus resistance). Although late blight-resistant varieties have already been developed and released by CIP, continuous breeding efforts are necessary since resistance typically breaks down a few years after the varieties are introduced into the field (Thiele et al. 2008).

Maximum rates of adoption for late blight-resistant varieties in the 32 target countries range from 10 to 60% and the expected yield gains range from 12 to 32%. This technology also entails cost reductions as a result of reduced use of fungicides. However, anecdotal evidence in some regions where late blight-resistant varieties are already cultivated reveals that farmers tend to keep spraying as if the varieties were susceptible. Therefore, the 5% cost reductions assumed reflect only a small proportion of the full potential that can be achieved in experimental trials. Since late blight-resistant varieties have been an important part of CIP’s research programs for a long time, a probability of success of 80% is given to this research option.

## 3 Model simulations

### 3.1 Simulating the adoption and the productivity impacts of new technologies

To simulate the effects of the selected crop productivity improvement technologies in the IMPACT model, different technologies can be defined at the FPU level. Each technology can occupy a specific share of the total production area dedicated every year to a particular crop in each FPU, and its adoption is modeled through the implementation of a logistic or linear diffusion curve which can be specified and parameterized prior to running the model. To simulate the yield advantage of a new technology over an old one, we appropriately modify the crop yield response functions included in the model. More precisely, in every FPU n, the yield equation for crop *j* and land type *k* (irrigated or rain-fed) for each year is expressed as:

(1) $$Y_{j,n,k}=YInt_{j,n,k}\times YInt2_{j,n,k}\times YWat_{j,n,k}\times YClim_{j,n,k}\times \left(PS_{j,c}\right)\varepsilon \times PF^{\eta }$$

where *YInt* is base year crop yield reported in FAOSTAT (in tons per ha), *YInt2* is an exogenous crop yield growth parameter, *YWat* is the exogenous yield shock from water stress (water availability), *YClim* is the exogenous yield shock from climate change (increased temperature), *PS* is the crop net price mapped to country *c*,^4^ e denotes the price response with respect to net price, *PF* represents prices of inputs, and g is the yield supply elasticity with respect to input prices. The impact from water and climate shocks on yields is estimated with the SUBSTOR potato growth model (Griffin et al. 1993) in the DSSAT (Decision Support System for Agrotechnology Transfer) farming system simulator (Jones et al. 2003), which is linked to the IMPACT modeling suite. All parameters in Equation (1) are specified as percentage changes over *YInt.* For example, when climate and water shocks are not considered, *YWat* and *YClim* take a value of one. More details about the yield equation and the how the different types of shock are defined in IMPACT can be found in Robinson et al. (2015).

The four potato technology options modeled here not only lead to changes in yield but also include changes in input costs and are contingent on the probability of success of the research efforts undertaken. In traditional economic surplus models, such as those proposed by Alston et al. (1998), the proportional shift in the supply curve (*k_t_* ) caused by a new technology in each target country at any year *t* is calculated as:

(2) $$k_{t}=\frac{\Delta Y}{\varepsilon_{s}}-\left(\frac{\Delta C}{1+\Delta Y}\right)\times p\times A_{t}$$

where Δ*Y* is the percentage maximum yield change, Ɛ_s_ is the price elasticity of supply, Δ*C* is the percentage input cost change, *p* is the probability of success in research, and *A_t_* is the adoption rate at year t, which is given by the logistic function:

(3) $$A_{t}=\frac{A_{max}}{1+e^{-\left(\alpha +\beta t\right)}}\;\text{for}\;t=1,2,...,T.$$

In the above equation, A_max_ is the maximum adoption rate, whereas α and β are parameters estimated using two points in the adoption curve, namely, *A_0_* and Amid which correspond to the initial and the 50% adoption rate occurring at years *t_0_* (year of initial adoption) and t_mid_, respectively.

Although *k_t_* adequately describes the supply shift in an economic surplus model, it cannot be applied in IMPACT because the supply shift itself is endogenously calculated when determining the market equilibrium and also incorporates feedback effects from other related commodity markets (cross-price effects). The same applies to the adoption rate and is specified prior to running the model. For these reasons, instead of a supply shift, we focus on modifying parameter *YInt2* in Equation (1) which represents a non-price-induced exogenous yield trend for a ‘baseline’ or ‘business as usual’ scenario of productivity growth. Any factor that modifies—additively or multiplicatively—the initial *YInt2* values corresponds to a scenario of accelerated growth in yields, such as that brought about by technological innovations from agricultural research. Given that the research-induced yield growth is conditional on the probability of success and needs to take into account possible cost changes, we calculated the percentage change of *YInt2* (denoted by A*YInt2)* with the following formula, which derives directly from the definition of *kt* in Equation (2):

(4) $$\Delta YInt2=\Delta Y-\left(\frac{\Delta C}{1+\Delta Y}\right)\times p.$$

By introducing an increase in productivity at the release year of the new technology, IMPACT simulates a new supply and demand equilibrium for the entire adoption period. In modeling terms, this one-time increase in productivity is sustained over time and translates to an upward shift of the projected yield growth over and above the growth which is already assumed by the model (expressed by *YInt2*). The difference between Equations (2) and (4) is that the former represents an outward shift in supply in economic surplus models, whereas Equation (4) represents an upward shift in the projected yield growth trend in IMPACT which finally leads to an endogenously calculated supply increase when running the model.

### 3.2 Foresight scenarios

To compare alternative approaches, one static and three foresight scenarios were specified for each of the four potato research options. The initial year of the simulations for all scenarios was 2015, and the length of the simulation period was 25 years, i.e. until 2040, which included the research and dissemination phases for each technology, as defined in Section 2.

The foresight scenarios derive from combinations of Representative Concentration Pathways (RCPs) and Shared Socioeconomic Pathways (SSPs) adopted by the IPCC. RCPs represent alternative trajectories on emissions and concentrations of greenhouse gases, and they are defined according to the radiative forcing projected by the end the 21st century (Van Vuuren et al. 2011). The climate projections from each RCP are performed with Earth System Models (ESMs) at various spatial and temporal resolutions and are used as data inputs for other applications, such as in crop modeling. SSPs are characterized by different challenges for mitigation and adaptation to climate change, and their quantification is based on assumed changes in income, population, and urbanization (among other variables) for most countries in the world (O’Neill et al. 2014).

The RCP-SSP combinations selected for this article draw on the compatibility matrix approach which has been proposed for developing scenarios in climate change analyses (Van Vuuren et al. 2014). More specifically, they are based on SSPs 1, 2, and 3 which represent pathways of low, medium, and high overall socioeconomic challenges, respectively. SSP1 is a ‘sustainability pathway’ with low population growth rates, high economic growth, and high levels of R&D investments that result in more environment-friendly technologies. SPP2 is a ‘middle of the road’ pathway that generally follows historic trends, while SSP3 is a more pessimistic scenario with higher population growth rates and more uneven economic growth (O’Neill et al. 2014). In this exercise, we consider SSP2 to be the benchmark pathway for which the initially assumed maximum adoption rates apply. To be more consistent with the assumptions of the different SSPs, *A*_max_ has been increased by 10% under SSP1 for all countries and technologies to account for higher R&D investments, whereas for SSP3 it was reduced by a similar percentage.

Each of the above SSPs can be combined with a different RCP to produce a consistent foresight scenario and to represent different levels of mitigation and adaptation costs. For this article, we selected RCP-SSP combinations that correspond to similar (medium) mitigation costs: SSP1-RCP4.5; SSP2-RCP6.0; and SSP3-RCP8.5.^5^ Among the three RCPs examined, RCP8.5 is the most extreme climate pathway, as it posits high green house gas emissions without mitigation policies, whereas RCP4.5 is a more optimistic pathway which assumes mitigation policies that lead to lower overall emissions. The same SSP-RCP combinations have also been used by Wiebe et al. (2015) in their analysis of the global climate change impacts on agriculture by 2050. Specific climate data for deriving the crop yield shock parameter *YClim* in Equation (1) with DSSAT were retrieved from the ESM model HadGEM2 (Hadley Centre Global Environment Model version 2) (Jones et al. 2011).

For the static scenario, we set a zero-growth rate to all parameters that change over time and are either linked to RCP and SSP assumptions (population, income, and climate-induced yield shocks) or are intrinsic to the model structure (e.g. exogenous yield growth). Thus, the static scenario approximated an economic surplus analysis with cross-price effects included in a multi-market structure.

When modeling multiple commodity markets, the literature suggests that welfare effects can be captured by examining the market in which the distortions appear, since the equilibrium will also reflect changes in all other vertically or horizontally related markets (Just et al. 2004). Therefore, to avoid double-counting (Alston et al. 1998), we estimated the expected welfare changes by focusing only on the potato market. Welfare changes from every technology in each scenario were estimated by comparing the outcomes against a baseline that involved an initial model run under the same scenario but without any productivity shifts. The baseline for the static scenario produced the same result for every year of the adoption period, since all dynamic elements of the model were held constant. The results were then aggregated (1) for all countries and (2) for target countries only, and we estimated the net present value (NPV) of the net welfare benefits (sum of consumer and producer surplus minus research and dissemination costs), the IRR, and the modified internal rate of return (MIRR) of each research option with both aggregation methods.^6^

## 4. Results

Table 2 reveals that the ordering of the different technologies in terms of net benefits is the same across country groups for all four scenarios, and it is also identical to what is reported in the original RTB study (Hareau et al. 2014); late blight-resistant varieties produce the highest welfare benefits, followed by virus resistance, bacterial wilt resistance, and improved seed systems.^7^ The ordering of the technologies according to their MIRR is slightly different, yet still consistent across all country groups and scenarios, with the virus-resistant varieties yielding the highest rate of return. In contrast, the ordering of the technologies according to their IRR is not the same between country groups, since improved seed systems produce a higher IRR than bacterial wilt-resistant varieties when only target countries are considered. The previous results suggest that the ordering of the different technologies, according to the three measures of investment performance used in this article, is independent of the foresight scenario examined, but it may be affected by the aggregation method employed.^8^

**Table 2:** Global economic impacts and benefit-cost results of potato research options under different scenarios

Benefit-cost indicators	*All countries*^a^				*Target countries*^a^			
	Static	SSP1-RCP4.5	SSP2-RCP6.0	SSP3-RCP8.5	Static	SSP1-RCP4.5	SSP2-RCP6.0	SSP3-RCP8.5
Improved seed systems								
Producer surplus	−264	−561	−504	−493	158	279	258	230
onsumer surplus	300	790	696	687	127	369	328	324
Net welfare benefits	−24	156	125	131	226	575	517	490
IRR	0.04	0.27	0.25	0.25	0.42	0.60	0.58	0.57
MIRR	0.07	0.16	0.15	0.17	0.20	0.26	0.25	0.25
Bacterial wilt-resistant varieties								
Producer surplus	−378	−916	−785	−782	198	352	317	262
Consumer surplus	529	1,450	1,240	1,253	202	686	586	592
Net welfare benefits	111	488	411	427	361	992	859	811
IRR	0.22	0.33	0.32	0.32	0.32	0.40	0.39	0.39
MIRR	0.17	0.23	0.23	0.23	0.22	0.27	0.26	0.26
Virus-resistant varieties								
Producer surplus	− 1,923	−3757	−3,212	−3,121	926	1,367	1,222	1,015
Consumer surplus	2,847	6,110	5,204	5,110	1,138	2,923	2,496	2,461
Net welfare benefits	892	2,316	1,957	1,957	2,032	4,253	3,684	3,444
IRR	0.70	0.92	0.88	0.88	0.98	1.18	1.14	1.12
MIRR	0.29	0.33	0.33	0.33	0.34	0.38	0.37	0.37
Late blight-resistant varieties								
Producer surplus	−2,868	−6,149	−5,366	−5353	1,202	1,813	1,672	1,373
Consumer surplus	4,187	9,584	8,366	8,420	1,640	4,610	4,020	4,061
Net welfare benefits	1,254	3,358	2,928	2,999	2,776	6,346	5,620	5,366
IRR	0.62	0.84	0.81	0.81	0.87	1.07	1.04	1.03
MIRR	0.27	0.32	0.31	0.32	0.32	0.36	0.36	0.35

a Results are calculated as changes over the baseline (IMPACT simulations for each SSP-RCP combination without the enhanced technologies); all welfare measures are expressed in NPVs, calculated with a discount rate of 10% and measured in million US$ at 2005 constant prices.

Although changes in consumer surplus are unambiguously positive for all technologies everywhere, potato producers in non-target countries suffer significant surplus losses because they face lower world prices without the benefit of higher yields. Lower prices are a disincentive for potato production and lead to decreases in yields and in land allocated to potato cultivation (Table 3). For target countries, the technology-induced supply increase is greater in effect than the decrease in world prices, leading to a positive producer surplus change. The difference in net benefits among country groups is particularly pronounced for the improved seed systems technology, and it is the principal reason for the inconsistent ordering of the different research options according to their IRR. Furthermore, improved seed systems are assumed to have short research and adoption lags, increased production costs, and the highest dissemination costs among all four technologies. These assumptions lead to the concentration of negative cash flows early in the beginning of the simulated 25-year period, thus yielding lower net benefits and IRR values compared to technologies with more uniformly distributed costs.

**Table 3:** Impacts of improved technologies on world potato production in 2040 (% change from baseline)

Production indicators	Improved seed systems	Bacterial wilt- resistant varieties	Virus-resistant varieties	Late blight- resistant varieties
Static scenario				
Yield				
*Target countries*	0.20	0.78	1.52	2.03
*Non-target countries*	−0.02	−0.11	−0.20	−0.29
*Global*	0.05	0.16	0.41	0.52
Land				
*Target countries*	0.04	0.23	0.25	0.49
*Non-target countries*	−0.05	−0.21	−0.39	−0.56
*Global*	−0.01	−0.02	−0.10	−0.07
Supply				
*Target countries*	0.24	1.01	1.77	2.53
*Non-target countries*	−0.07	−0.31	−0.59	−0.84
*Global*	0.04	0.18	0.31	0.44
SSP1-RCP4.5				
Yield				
*Target countries*	0.24	0.86	1.43	2.31
*Non-target countries*	−0.04	−0.15	−0.24	−0.41
*Global*	0.07	0.25	0.48	0.74
Land				
*Target countries*	0.03	0.23	0.18	0.40
*Non-target countries*	−0.07	−0.30	−0.48	−0.79
*Global*	−0.02	−0.03	−0.12	−0.14
Supply				
*Target countries*	0.27	1.09	1.61	2.72
*Non-target countries*	−0.11	−0.45	−0.72	−1.20
*Global*	0.05	0.22	0.36	0.61
SSP2-RCP6.0				
Yield				
*Target countries*	0.24	0.80	1.23	2.11
*Non-target countries*	−0.04	−0.14	−0.21	−0.37
*Global*	0.07	0.23	0.39	0.66
Land				
*Target countries*	0.03	0.21	0.19	0.38
*Non-target countries*	−0.04	−0.27	−0.41	−0.71
*Global*	−0.02	−0.03	−0.08	−0.11
Supply				
*Target countries*	0.26	1.01	1.42	2.50
*Non-target countries*	−0.10	−0.41	−0.62	−1.07
*Global*	0.05	0.20	0.31	0.54
SSP3-RCP8.5				
Yield				
*Target countries*	0.20	0.70	1.05	1.89
*Non-target countries*	−0.03	−0.13	−0.20	−0.36
*Global*	0.07	0.23	0.38	0.68
Land				
*Target countries*	0.02	0.16	0.14	0.28
*Non-target countries*	−0.06	−0.26	−0.39	−0.69
*Global*	−0.02	−0.04	−0.09	−0.15
Supply				
*Target countries*	0.23	0.87	1.19	2.18
*Non-target countries*	−0.10	−0.39	−0.58	−1.05
*Global*	0.05	0.20	0.29	0.53

The static scenario also produces negative net benefits for the improved seed systems technology when all countries are considered. Although from an investment viewpoint this result may suggest that improved potato seed systems, as specified in this scenario, are not a reasonable research option, it should be viewed with caution, as it contradicts common agronomic knowledge that low-quality seed constrains the expression of the crop’s full yielding ability (Haverkort and Struik 2015). In fact, negative net benefits are a direct consequence of the underestimation of the welfare impacts of all technologies under the static scenario. More precisely, Table 2 shows that the disparity in net benefits is mainly driven by consumer surplus, which in the static scenario is less than half of those seen in each of the foresight scenarios and can be explained by the static demand which ignores the expected growth in population and income. Similar results are also observed on the production side, namely, for potato yields, harvested land, and total supply in non-target countries, for which the static scenario generally underestimates the negative impacts of the new potato technologies (Table 3). As a consequence, it leads to smaller producer surplus losses compared to the foresight scenarios when all countries are considered.

All technology alternatives analyzed in this article demonstrate higher areas of adoption under SSP3-RCP8.5 and consequently exhibit higher dissemination costs (Table 4). Since the adoption rates for each technology are exogenously defined as a percentage of the total potato harvested area, this result also implies that the area allocated to potato in target countries under SSP3-RCP8.5 is higher than in other scenarios. The same scenario also yields the highest net benefits for all technologies, except for virus-resistant varieties which produce higher returns under SSP1-RCP4.5. More specifically, RCP8.5 is the driest climate change scenario among the three examined and consequently leads to lower overall yields and smaller producer surplus changes in target countries compared to the other two scenarios. At the same time, SSP3 represents the highest population growth among all SSPs (but also the slowest income growth) and thus results in higher demand for staple agricultural commodities. These assumptions are reflected in the higher consumer surpluses observed under SSP3-RCP8.5. In contrast, SSP1-RCP4.5 represents a less rapid climate change scenario^9^ and assumes the lowest population increase among all SSPs, thus leading to opposite impacts than SSP3-RCP8.5.

**Table 4:** Adoption of improved potato varieties

Adoption indicators	Improved seed systems	Bacterial wilt-resistant varieties	Virus-resistant varieties	Late blight-resistant varieties
Dissemination costs (million US$)^a^				
Static scenario	37.7	12.8	16.6	35.1
SSP1-RCP4.5	50.9	19.3	21.5	46.4
SSP2-RCP6.0	46.2	17.4	19.3	41.9
SSP3-RCP8.5	42.2	16.0	17.6	38.3
Total costs (million US$)^a^				
Static scenario	59.6	39.9	31.9	65.6
SSP1-RCP4.5	72.8	46.4	36.7	77.0
SSP2-RCP6.0	68.0	44.4	34.6	72.5
SSP3-RCP8.5	64.1	43.0	32.9	68.9
Total adoption area (thousand hectares)				
Static scenario	774.4	1,020.8	618.0	1,303.3
SSP1-RCP4.5	1,044.0	1,614.4	827.5	1,798.8
SSP2-RCP6.0	963.8	1,445.3	742.3	1,617.7
SSP3-RCP8.5	865.8	1,330.4	675.4	1,483.4

a Reported results are NPVs expressed at 2005 constant prices and calculated with a discount rate of 10%.

Although our results reveal substantial differences in the net benefits among the various scenarios, the parameters used in the specification of the technologies lead to rather limited supply growth in target countries, which ranges from 0.23% (improved seed systems—SSP3-RCP8.5) to 2.72% for the late blight resistance technology in the SSP1-RCP4.5 scenario (Table 3). Except for China and India, target countries are responsible for only a small share of the global potato production, and therefore the resulting decrease in world prices is also small and estimated at around 0.1-1.8%, depending on the technology and the scenario examined (Table 5). The above changes in the potato sector are also expected to alter the allocation of land among different activities, shift total supply, and modify the clearing prices for other agricultural commodities. The new equilibrium is determined by changes in relative prices (substitution effects) and the increase in available income due to a fall of consumer expenditures on potato as a result of its lower price. Table 5 also presents the world price changes for rice and wheat, which are crops commonly related to potato either in consumption (rice in Asia) or in production (potato and winter cereals are grown at the same time of the year, although they do not necessarily compete for the same land). Our results show that the price feedback effects are small, which can be explained by the relatively smaller size of the potato sector compared to cereals that dominate agricultural production in target countries.^10^ Therefore, the role of foresight on cross-price effects depends on the relative magnitude of the examined intervention.

**Table 5:** Impacts of potato technologies on world producer prices of potato and related commodities (% change from baseline)

Commodities	Improved seed systems	Bacterial wilt-resistant varieties	Virus-resistant varieties	Late blight-resistant varieties
Static scenario				
Potato	−0.11	−0.48	−0.91	−1.30
Rice	^a^	^a^	−0.01	−0.01
Wheat	^a^	^a^	−0.01	−0.01
SSP1-RCP4.5				
Potato	−0.16	−0.66	−1.07	−1.78
Rice	^a^	^a^	−0.01	−0.01
Wheat	^a^	−0.01	−0.01	−0.01
SSP2-RCP6.0				
Potato	−0.15	−0.60	−0.92	−1.59
Rice	^a^	^a^	−0.01	−0.01
Wheat	^a^	^a^	−0.01	−0.01
SSP3-RCP8.5				
Potato	−0.14	−0.58	−0.86	−1.55
Rice	^a^	^a^	−0.01	−0.01
Wheat	^a^	−0.01	−0.01	−0.01

a Lower than —0.01%.

## 5. Discussion

Our analysis reveals that introducing dynamics in the form of foresight scenarios in this priority setting exercise does not affect the ranking of the examined technologies according to their expected net benefits or either measure of rate of return to investment. In fact, the ordering of the different potato research options is identical to the original RTB study which was based on a simple economic surplus model. Therefore, foresight considerations in assessing research options become irrelevant in this case, if the objective is simply to provide an ordinal comparison of the returns from investing in different research options. However, foresight scenarios greatly affect the estimated absolute level of welfare impacts. The discrepancy in the estimated net benefits between the static and the foresight scenarios also suggests that simple economic surplus models may not always be appropriate for setting priorities and allocating resources because such analyses typically rely on the NPV of net benefits of each candidate activity. Given that the welfare impacts of each examined technology vary significantly among the three foresight scenarios, there seems to be scope in including foresight considerations in priority setting exercises depending on the research objective, especially when the uncertainty about climate change needs to be explicitly accounted for.

Another aspect of the analysis sheds light on the shortcomings of the use of single market economic surplus models in priority setting, in which welfare impacts are typically calculated for target countries only. According to our results, ignoring non-target countries leads to an overestimation of net benefits by an order of almost two in all simulation scenarios. This finding highlights the ‘accuracy versus cost’ trade-off that economists face when carrying out such exercises, which require the collection and consolidation of data and information from various sources and across different regions and countries. It also raises concerns because priority setting studies in international agricultural research do not always consider nontarget countries in their analysis. This may also have important implications for resource allocation, not only among different research activities but also among different social purposes in different world regions. Notwithstanding the obvious challenges for modeling the global agricultural sector, our results clearly show that priority setting studies using economic surplus models should nonetheless account for the global welfare impacts of the examined interventions, even if this can only be done aggregately by assuming a single market for the rest of the world. The previous postulate applies to any objective used in a priority setting study and not just to net benefits. For example, nutritional security and poverty reduction have not been considered in the present analysis, but they usually rank high in policy agendas and are key determinants of resource allocation. The consequences of a new agricultural technology on these two objectives will not be limited to target countries only but will bring about global changes in food supply which must be also taken into account.

The impact of the new potato technologies on other related commodities has also been found to be very small, which implies that the single-commodity focus of the simple economic surplus model is an acceptable assumption for the present analysis, given how the examined potato technologies were specified. However, the added value of a multi-commodity approach, compared to the simple economic surplus model, depends on the extent to which one expects cross-price effects. The added value will therefore be small for an innovation that affects a small or isolated market but can prove important for an innovation that affects a large market with close links across countries and commodities. The major drawback of the single-commodity model concerns the assessment of impacts related to objectives that span across multiple commodity markets and cannot be well represented by price changes alone. For instance, even if individual cross-price effects are small (thus leading to similarly small changes in supply), the assessment of non-economic impacts, like nutritional security, still requires accounting for supply changes in all agricultural commodities. Therefore, the objectives and criteria included in a priority setting study determine the validity of the single-commodity assumption, even in cases where the cross-price effects of the examined interventions are expected to be limited.

All priority setting exercises are carried out in an *ex ante* framework and make use of a set of assumptions regarding adoption and productivity gains. As in the case of the original RTB study, values for these parameters may come from various sources, which are usually qualitative in nature, and complement quantitative approaches that can make more assumptions explicit and examine their validity and importance in ranking research priorities. However, results are also conditional on the validity of the assumptions regarding the realization of the foresight scenarios, the calculation of the different measures of investment performance, and the representation of the global multi-market equilibrium with the IMPACT model. For example, we have assumed different maximum adoption rates for the three SSPs to account for the different underlying socioeconomic conditions that may affect technology dissemination and adoption. However, modeling results not reported in this article reveal that the ordering of the technologies remains unaffected even if the maximum adoption rates for each technology are instead kept constant across all SSPs.

We have implicitly assumed that there are no technological spillovers between countries and regions; yet such indirect effects are possible and have already attracted much attention in the relevant literature. Technological spillovers could mitigate partially—or fully—the negative impacts on potato production in non-target countries but could also reduce the positive impacts in target countries. In either case, the direction of changes in producer surplus would still depend on the relative magnitude of productivity gains and price decreases. Introducing spatial technology spillovers in IMPACT is straightforward and may be part of future work that also extends the present analysis to other commodities.

Another set of assumptions concerns the numerical value of some key parameters in the IMPACT model which are subject to uncertainty. For example, we have introduced a productivity shift by modifying the exogenous yield growth rates in the model, which themselves are estimated from historical trends and adjusted according to expert judgment on regional potential for development of different commodities. We have used a single ESM to quantify the different RCPs in terms of yield losses, although other ESMs would probably produce different welfare results. Past experience, however, has shown that scenarios of alternative technologies usually give consistent results across both RCPs and SSPs in terms of the direction and relative magnitude of impacts. The size, complexity, and deterministic nature of IMPACT do not easily allow in-depth examination of model solution robustness across a broad set of parameters. For this reason, the definition of multiple consistent foresight scenarios can also serve as a sensitivity analysis exercise which can produce a band of plausible welfare outcomes for testing some key modeling assumptions as a means to improve our understanding of the role of selected dynamic parameters in a foresight study.

Our analytical framework does not capture all possible welfare effects because it does not provide any information about possible impacts on other economic sectors outside of agriculture, for example the starch processing industry. Further work with general equilibrium approaches is required to examine the economy-wide effects of introducing new crop technologies and to test whether such elaborate models can better inform priority setting exercises. The shortcoming of computable general equilibrium (CGE) models is that they usually include an aggregate representation of the agricultural sector which makes them unsuitable for examining crop- specific technologies. In contrast, sector equilibrium models like IMPACT can distinguish between multiple crop and livestock activities and are thus able to capture spillover price effects across different commodity markets and regions, despite ignoring welfare consequences on other economic sectors. A solution to overcome the individual limitations of both types of models is to use them jointly in a sequential way. Hence, the results of agricultural sector models could be used as inputs in CGE models to estimate economy-wide impacts. An example is the effort to couple IMPACT with CGE models to tackle issues like poverty alleviation, which is difficult to answer when only focusing on the agriculture sector (Robinson et al. 2015).

## 6. Conclusions

Resource allocation depends on the relative importance that decision makers and donors attach to their funding objectives but also on the available information regarding the expected impacts of research. In this article, we show how economic surplus analyses of the benefits of agricultural technologies can be incorporated in global dynamic multi-commodity models (specifically the IMPACT model). We argue that the assessment of research priorities which considers dynamics in the form of foresight scenarios may offer valuable insights on how possible biophysical and socioeconomic changes may affect the welfare impacts of a given intervention. We also show that limiting the analysis to target countries should be avoided, as it may lead to an overestimation of the expected welfare changes. On the other hand, the added value from the multi-market analysis seems to be low when the examined interventions have only small impacts on related commodity markets.

These findings suggest that the assumptions underpinning an economic surplus model can seriously affect the estimation of the expected welfare impacts of the examined interventions. For international agricultural research, in particular, where new technologies target multiple countries and can have important direct and indirect welfare impacts, global dynamic multi-commodity models provide a more informative alternative to the economic surplus model approach. For this reason there is scope for including them in priority setting studies, although they come at additional cost; their complexity and their large set of assumptions for the representation of the global agricultural sector and the quantification of foresight scenarios may be a limiting factor for their wider use, especially when considering the simplicity of the single-commodity economic surplus model approach which provides a cost-effective way to implement priority setting in times of tightened budgets for international agricultural research. Improving the validity of these assumptions through better parameterization and stronger multidisciplinary collaboration is therefore necessary for increasing the usefulness of such models in informing research prioritization and decision-making.

## Notes

1. More information about how the different surplus measures are calculated with IMPACT’s welfare module can be found in Robinson et al. (2015).

2. The RTB exercise identified two more research options, namely, the ‘Development of potato value chains’ and the ‘Intensification of cereal-based potato systems: the *agile potato*’. These technologies are not considered in this article to simplify the analysis.

3. This section and the description of the potato research options draw on the original RTB priority assessment exercise (Hareau et al. 2014).

4. The price parameters are country-specific and thus apply for all FPUs in the same country.

5. The number accompanying the selected RCPs (e.g. 8.5) represents the radiative forcing assumptions for 2100 (in Watts per m^2^), and can be interpreted as the intensity of the assumed change in climate.

6. Although the MIRR was not reported in the original RTB study, we calculate it in this article to address some of the criticism related to the use of IRR as an indicator of profitability. Specifically, the IRR assumes that the intermediate cash flows are reinvested at a discount rate equal to the IRR itself, which is considered unrealistic. In contrast, the calculation of the MIRR is based on the use of socially acceptable discount rates for borrowing and investing and leads to lower, albeit more plausible, rates of return compared to the IRR. A very comprehensive discussion about the mathematical properties, the underlying assumptions and the suitability of the IRR and MIRR for the evaluation of R&D investments can be found in Hurley et al. (2014, 2016) and Oehmke (2016).

7. The original RTB study calculated the NPV of the expected welfare benefits with discount rates of 5 and 10%. To simplify the exposition of the results, in this article we only use a discount rate of 10%. However, simulation results not reported here due to space limitations reveal that the technology ordering does not change when a 5% discount rate is assumed instead.

8. Priority setting by comparing the rate of returns seldom makes sense as IRRs, and MIRRs do not consider the size effects that are shown with NPVs. The reason for presenting the IRR- and MIRR-based ranking is to identify differences between the modeling assumptions used (result aggregation between target countries versus all countries).

9. Although RCP6.0 implies higher radiative forcing than RCP4.5 by 2100, during our simulation period (until 2040) RCP6.0 corresponds to a milder climate change pathway.

10. On average for 2012-14, harvested potato areas in target countries amount at about 8.5% of rice areas and 13.5% of wheat areas (FAO 2017).
