# Supplementary material for: Comparing modeling approaches for assessing priorities in international agricultural research
Source: Res Eval. 2019 Sep 17;27(2):145–56. doi: 10.1093/reseval/rvx044 (PMC7771476; doi:10.1093/reseval/rvx044)
Supplement: Supplementary file 1 [file RE-27-02-145-s001.docx]

**Analytical data for each potato research option**

Table A: Parameter values for improved seed systems.

| Country | Maximum adoption rate (% of total area) | Year of first adoption | Adoption lag (years) | Yield increase (%) | Cost change (%) | Probability of success (%) |
| --- | --- | --- | --- | --- | --- | --- |
| Peru | 7% | 3 | 9 | 20% | 20% | 70% |
| Ecuador | 7% | 3 | 5 | 20% | 20% | 70% |
| Bolivia | 3% | 3 | 5 | 20% | 20% | 70% |
| Colombia | 3% | 3 | 5 | 20% | 20% | 70% |
| China (Guizhou) | 20% | 3 | 5 | 20% | 20% | 60% |
| China (Yunnan) | 20% | 3 | 5 | 20% | 20% | 60% |
| China (Sichuan, Chongqing) | 20% | 3 | 5 | 20% | 20% | 60% |
| Philippines | 20% | 3 | 5 | 20% | 20% | 60% |
| Uganda | 10% | 3 | 5 | 20% | 20% | 70% |
| Nigeria | 8% | 3 | 5 | 20% | 20% | 70% |
| Congo, DRC | 7% | 3 | 5 | 20% | 20% | 70% |
| Tanzania | 7% | 3 | 5 | 20% | 20% | 75% |
| Madagascar | 8% | 3 | 5 | 20% | 20% | 70% |
| Burundi | 10% | 3 | 5 | 20% | 20% | 70% |
| Rwanda | 20% | 3 | 5 | 20% | 20% | 70% |
| Angola | 2% | 3 | 5 | 20% | 20% | 70% |
| Malawi | 13% | 3 | 5 | 20% | 20% | 70% |
| Mozambique | 13% | 3 | 5 | 20% | 20% | 70% |
| Ethiopia | 13% | 3 | 5 | 20% | 20% | 70% |
| Kenya | 20% | 3 | 5 | 20% | 20% | 80% |
| Cameroon | 7% | 3 | 5 | 20% | 20% | 70% |
| India | 20% | 3 | 5 | 20% | 20% | 70% |
| Bhutan | 20% | 3 | 5 | 20% | 20% | 60% |
| Uzbekistan | 20% | 3 | 5 | 20% | 20% | 60% |
| Armenia | 20% | 3 | 5 | 20% | 20% | 60% |
| Tajikistan | 20% | 3 | 5 | 20% | 20% | 60% |
| Georgia | 20% | 3 | 5 | 20% | 20% | 60% |

Table B: Parameter values for bacterial wilt resistant varieties.

| Country | Maximum adoption rate (% of total area) | Year of first adoption | Adoption lag (years) | Yield increase (%) | Cost change (%) | Probability of success (%) |
| --- | --- | --- | --- | --- | --- | --- |
| Bolivia | 18% | 10 | 10 | 20% | 0 | 50% |
| Colombia | 30% | 10 | 10 | 10% | 0 | 50% |
| China (Yunnan) | 30% | 10 | 10 | 20% | 0 | 50% |
| China (Sichuan, Chongqing) | 30% | 10 | 10 | 20% | 0 | 50% |
| Philippines | 20% | 10 | 10 | 10% | 0 | 50% |
| Uganda | 40% | 10 | 10 | 30% | 0 | 50% |
| Nigeria | 10% | 10 | 10 | 30% | 0 | 50% |
| Congo, DRC | 20% | 10 | 10 | 30% | 0 | 50% |
| Tanzania | 20% | 10 | 10 | 30% | 0 | 50% |
| Madagascar | 10% | 10 | 10 | 30% | 0 | 50% |
| Burundi | 40% | 10 | 10 | 30% | 0 | 50% |
| Rwanda | 60% | 10 | 10 | 30% | 0 | 50% |
| Ethiopia | 40% | 10 | 10 | 30% | 0 | 50% |
| Kenya | 40% | 10 | 10 | 30% | 0 | 50% |
| Cameroon | 10% | 10 | 10 | 30% | 0 | 50% |
| India (Bihar) | 60% | 10 | 10 | 10% | 0 | 50% |
| Nepal | 20% | 10 | 10 | 10% | 0 | 50% |
| Bangladesh | 20% | 10 | 10 | 20% | 0 | 50% |
| Bhutan | 20% | 10 | 10 | 10% | 0 | 50% |
| India (West Bengal) | 60% | 10 | 10 | 10% | 0 | 50% |
| Pakistan | 20% | 10 | 10 | 20% | 0 | 50% |

Table C: Parameter values for virus resistant varieties.

| Country | Maximum adoption rate (% of total area) | Year of first adoption | Adoption lag (years) | Yield increase (%) | Cost change (%) | Probability of success (%) |
| --- | --- | --- | --- | --- | --- | --- |
| China (Gansu) | 20% | 2 | 10 | 40% | -5% | 70% |
| China (Qinghai) | 20% | 2 | 10 | 40% | -5% | 70% |
| China (Ningxia) | 20% | 2 | 10 | 40% | -5% | 70% |
| China (Nei Mongol) | 20% | 2 | 10 | 40% | -5% | 70% |
| Uzbekistan | 40% | 2 | 10 | 40% | -5% | 70% |
| Armenia | 35% | 2 | 10 | 40% | -5% | 70% |
| Tajikistan | 30% | 2 | 10 | 40% | -5% | 70% |
| Kazakhstan | 35% | 2 | 10 | 40% | -5% | 70% |
| Kyrgyzstan | 35% | 2 | 10 | 40% | -5% | 70% |
| Kenya | 35% | 2 | 10 | 40% | -5% | 70% |
| Rwanda | 55% | 2 | 10 | 40% | -5% | 70% |
| Mozambique | 5% | 2 | 10 | 40% | -5% | 70% |
| Malawi | 5% | 2 | 10 | 40% | -5% | 70% |
| Tanzania | 15% | 2 | 10 | 40% | -5% | 70% |
| Uganda | 35% | 2 | 10 | 40% | -5% | 70% |
| Angola | 5% | 2 | 10 | 40% | -5% | 70% |
| Ethiopia | 5% | 2 | 10 | 40% | -5% | 70% |
| Burundi | 35% | 2 | 10 | 40% | -5% | 70% |
| Cameroon | 5% | 2 | 10 | 40% | -5% | 70% |
| Madagascar | 5% | 2 | 10 | 40% | -5% | 70% |
| Nigeria | 5% | 2 | 10 | 40% | -5% | 70% |

Table D: Parameter values for late blight resistant varieties.

| Country | Maximum adoption rate (% of total area) | Year of first adoption | Adoption lag (years) | Yield increase (%) | Cost change (%) | Probability of success (%) |
| --- | --- | --- | --- | --- | --- | --- |
| Peru | 40% | 2 | 10 | 32% | -5% | 80% |
| Ecuador | 40% | 2 | 10 | 32% | -5% | 80% |
| Bolivia | 18% | 2 | 10 | 32% | -5% | 80% |
| Colombia | 30% | 2 | 10 | 32% | -5% | 80% |
| China (Guizhou) | 30% | 2 | 10 | 16% | -5% | 80% |
| China (Yunnan) | 30% | 2 | 10 | 16% | -4% | 80% |
| China (Sichuan, Chongqing) | 30% | 2 | 10 | 16% | -4% | 80% |
| Myanmar | 10% | 2 | 10 | 32% | -5% | 80% |
| Vietnam | 30% | 2 | 10 | 16% | -5% | 80% |
| Philippines | 20% | 2 | 10 | 32% | -5% | 80% |
| Indonesia | 10% | 2 | 10 | 32% | -5% | 80% |
| Uganda | 40% | 2 | 10 | 24% | -5% | 80% |
| Nigeria | 10% | 2 | 10 | 24% | -5% | 80% |
| Congo, DRC | 20% | 2 | 10 | 24% | -5% | 80% |
| Tanzania | 20% | 2 | 10 | 24% | -5% | 80% |
| Madagascar | 10% | 2 | 10 | 24% | -5% | 80% |
| Burundi | 40% | 2 | 10 | 24% | -5% | 80% |
| Rwanda | 60% | 2 | 10 | 24% | -5% | 80% |
| Angola | 10% | 2 | 10 | 24% | -5% | 80% |
| Malawi | 10% | 2 | 10 | 24% | -5% | 80% |
| Mozambique | 10% | 2 | 10 | 24% | -5% | 80% |
| Ethiopia | 40% | 2 | 10 | 24% | -5% | 80% |
| Kenya | 40% | 2 | 10 | 24% | -5% | 80% |
| Cameroon | 10% | 2 | 10 | 24% | -5% | 80% |
| Nepal | 20% | 2 | 10 | 32% | -5% | 80% |
| Bangladesh | 20% | 2 | 10 | 16% | -5% | 80% |
| Bhutan | 20% | 2 | 10 | 32% | -5% | 80% |
| India (West Bengal) | 60% | 2 | 10 | 16% | -2% | 80% |
| Pakistan | 20% | 2 | 10 | 16% | -4% | 80% |
| Armenia | 40% | 2 | 10 | 12% | -4% | 80% |
| Azerbaijan | 40% | 2 | 10 | 12% | -4% | 80% |
| Uzbekistan | 60% | 2 | 10 | 12% | -4% | 80% |
| Tajikistan | 36% | 2 | 10 | 12% | -4% | 80% |
| Georgia | 40% | 2 | 10 | 12% | -4% | 80% |
